# Hypnotic suggestions of safety reduce neuronal signals of delay discounting

**DOI:** 10.1038/s41598-021-81572-2

**Published:** 2021-02-01

**Authors:** Barbara Schmidt, Clay B. Holroyd

**Affiliations:** 1 Institute of Psychology, University of Jena, Am Steiger 3, Haus 1, 07743 Jena, Germany; 2 Department of Experimental Psychology, Ghent University, Ghent, Belgium

**Keywords:** Human behaviour, Neuroscience, Psychology

## Abstract

Waiting for delayed rewards is important to reach long-term goals, yet most people prefer immediate rewards. This tendency is called delay discounting. Evidence shows that people are more willing to wait for delayed rewards when they believe that the delayed reward is certain. We hypothesized that feeling safe makes delayed outcomes subjectively more certain, which should in turn reduce neuronal signals of delay discounting. We hypnotized 24 highly suggestible participants and gave them a suggestion to feel safe. We then used EEG to measure their brain responses to immediate and delayed rewards while they played a delayed gratification game. As compared to a control condition without hypnosis, participants that were suggested to feel safe under hypnosis reported feeling significantly safer. Further, their reward-related brain activity differentiated less between immediate and delayed rewards. We conclude that feeling safe makes delayed outcomes subjectively more certain and therefore reduces neuronal signals of delay discounting.

## Introduction

When deciding between two immediate rewards, most people reasonably prefer a larger reward over a smaller reward. Yet when the choice is between an immediate reward and a larger reward that is delayed in time, many people switch their preference for the smaller reward. The decrease in value of the reward as function of delay is called delay discounting^1^. It seems that in order to wait for delayed rewards, we must override our natural tendency to devalue them. For example, a series of famous experiments by Walter Mischel and his colleagues drew attention to the association between individual differences in delay discounting and positive long-term outcomes^2–4^. Preschool children were required to decide between either receiving one marshmallow immediately or waiting several minutes for two marshmallows. The children who waited in this task showed better grades at school, better social competence and higher ambitions^2–4^. The observed correlations between performance in the marshmallow task and behavioral measures of impulsivity and self-control persisted even 40 years after the initial test^5^. By contrast, individuals with disorders accompanied by low levels of self-control and high levels of impulsivity, such as drug addiction and gambling disorders, show increased delay discounting^6–8^.

So, what is it that makes waiting for delayed rewards so difficult? Note that immediate rewards are relatively certain, whereas delayed rewards are relatively uncertain, as any number of unforeseen events could transpire between the present and the time of reward delivery. Delayed rewards therefore require estimates of the waiting duration and of the certainty of reward delivery. Participants in delay discounting experiments are hence more likely to wait for rewards that they believe are likely to be delivered despite the wait^9^. Further, family background and home environment – where safe environments foster feelings of stability and reliability – strongly contribute to the association between toddler performance in the marshmallow task and subsequent adolescent achievement^10^. Also, state factors affect perceptions of certainty about delayed reward. In an early study by Alvin Mahrer^1^, an experimenter promised to bring young schoolchildren a balloon the next day. The experimenter kept this promise in a *reliable* group but did not in an *unreliable* group. Subsequently, when the children were asked to decide between a toy now or a better toy tomorrow, the reliable group chose the better toy more often than did the unreliable group. A similar study by Celeste Kidd and colleagues also showed that children were more willing to wait when they believed the experimenter to be trustworthy^11^. The experimenter’s trustworthiness can be considered a state indicator of increased subjective probability that a delayed reward will be delivered. With repetition, this state effect could become a trait effect associated with reliable family background and safe home environment.

Based on these results, we hypothesized that an experimentally induced feeling of safety would increase the preference for delayed rewards. We tested this hypothesis by inducing a state-dependent feeling of safety in highly suggestible participants and then recording the electroencephalogram (EEG) while they engaged in a delayed gratification task. Toward this end, we told participants to imagine that they were in a safe place^12^. To enhance the impact of this suggestion, we hypnotized the participants before giving the suggestion. Hypnosis has been defined as “a state of consciousness involving focused attention and reduced peripheral awareness characterized by an enhanced capacity for response to suggestion”^13^. Suggestions work better when participants are hypnotized^14^, and positive suggestions during hypnosis can reduce anxiety and stress in medical contexts^15,16^. The effectiveness of hypnotic interventions has been shown in meta-analyses on hypnotic analgesia^17,18^, hypnotic interventions during medical procedures^15^ and for hypnosis as an adjunct to cognitive-behavioral therapy^19^. Therefore, we combined hypnosis with the suggestion of being at a safe place to induce robust feelings of safety, and then investigated how this feeling of safety affects behavior and reward-related brain activity in a delayed gratification game.

To be specific, we examined a performance-related EEG response that shows a negative amplitude around 250 ms following negative outcomes^20^. The reward positivity is a difference wave capturing the difference between negative and positive monetary outcomes^21–23^. A larger difference is associated with higher reward-sensitivity^24,25^. The reward positivity shows high test–retest reliability, so we assume that it reflects a stable personality trait^22,26,27^. Importantly in the context of our current study, the reward positivity has been strongly implicated in disorders related to impulsivity such as addiction and ADHD^21,28–31^.

In the context of delayed rewards, high delay discounting is associated with larger reward positivity amplitudes for immediate rewards as compared to low delay discounting, suggesting that high discounters overvalue immediate rewards^32^. In a recent delay discounting study, we aggregated over reward magnitude to compare immediate and delayed rewards directly^27^, and found that participants who scored high on impulsivity and low on self-control showed higher reward positivity amplitudes^27^. That means that participants with larger differences between brain responses to immediate and delayed rewards – what we termed the “delay reward positivity” – tended to be more impulsive and less self-controlled.

In the current study, participants played the delayed gratification game as reported in Schmidt et al.^27^ according to a within-subject experimental design with two conditions. In a hypnosis condition, prior to them playing the game we hypnotized highly suggestible participants and asked them to imagine being at a safe place. In a control condition, the same participants played the game without hypnosis and the suggestion of safety. We expected that when they felt relatively safe, the participants’ brain responses would differentiate less between immediate and delayed rewards as compared to the control condition, indicating less impulsive behavior.

## Method

### Participants

In previous hypnosis studies conducted in our laboratory, the within-subjects effect size for event-related EEG potentials was *d* = 0.7^33,34^. With a power level of 0.95 and an alpha level of 0.05, 24 participants are required according to G*power^35^. Therefore, we collected data from 24 participants (12 female) whose mean age was 25.2 years (range 19–40 years). These participants were selected from a pool of subjects who were previously pre-tested in a separate experimental session for their level of suggestibility using the Harvard Group Test of Hypnotic Susceptibility (HGSHS^36^). In the HGSHS, the experimenter hypnotizes a group of participants and then presents 12 suggestions. Dependent on the number of suggestions participants successfully complete, they are assigned a score of 0 to 12. For our study, we invited participants with suggestibility scores of at least 8 out of 12 (M = 8.6, range 8–11), indicating high suggestibility. Participants were paid according to the outcomes in the delayed gratification game and in a risk game that are described elsewhere^37^. The average payment that was paid out immediately was 23.5 Euro (SD = 0.2 Euro). This sum contains the immediate rewards of the delayed gratification game, which was 6.9 Euro (SD = 0.1 Euro), and the outcome of the other economic paradigm. The delayed gratification game also contained rewards that were paid out six months later. The average payment six months later was 6.9 Euro (SD = 0.1 Euro). The study was carried out in accordance with the Declaration of Helsinki and was approved by the ethics committee of the Friedrich Schiller University of Jena.

### Apparatus

The experimental tasks were programmed and presented in Presentation software (Neurobehavioral Systems, Inc., Berkeley, CA, www.neurobs.com). Statistical analyses were computed with R^38^. To compute within-subject effect sizes, we used Cohen’s *d* according to the formula provided by Lakens^39^ in Eq. 7. For ANOVA within-subject effect sizes, we used generalized eta squared as recommended by Bakeman^40^.

### Procedure

**Figure 1:**
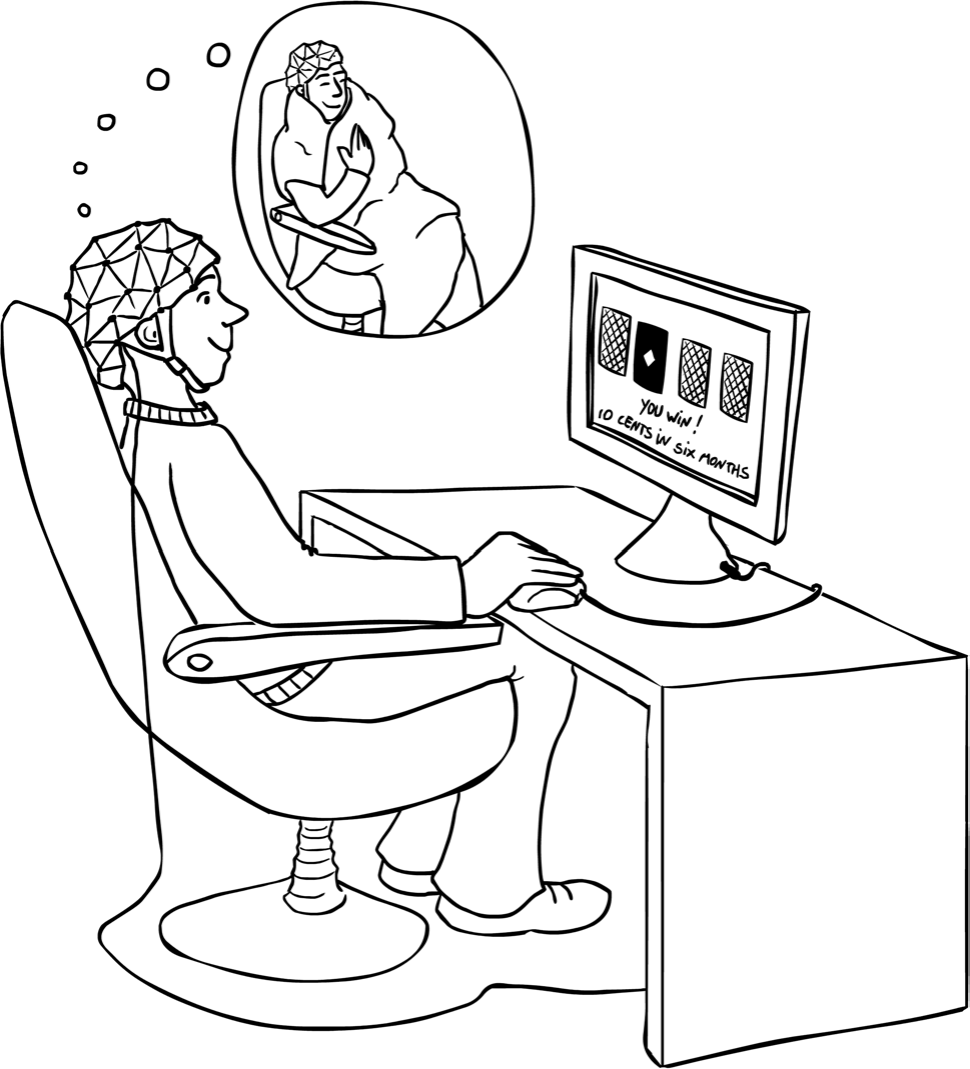
Illustration of the suggestion of safety involving an image of being cuddled into a blanket. This figure was drawn by Anne Rasch https://annekarenrasch.blogspot.com/.

Participants read a participant information sheet including the description of the delayed gratification task and provided informed consent in the beginning of the experiment. The experimenter showed a paper version of the playing cards that would occur in the game later and explained their meaning once again. Then, an electrode cap with 64 electrodes (EASYCAP, Woerthsee-Etterschlag, Germany) for recording the EEG was placed on the participants’ head. Participants were seated in a dimly lit room on a comfortable chair, approximately 100 cm in front of a computer monitor. The experimenter communicated with the participants via a microphone sitting outside the EEG chamber while the participants wore in-ear headphones inside the EEG chamber. The participants could not see the experimenter during the hypnosis and control conditions. The experimenter observed the participants via two cameras: the first showing the participants in full view and the second showing the participants’ faces in close up. Participants played the delayed gratification task after a risk game that is reported elsewhere^37^, in both a hypnosis condition and a control condition, the order of which was counter-balanced across participants. In the hypnosis condition, the experimenter conducted a hypnosis induction according to the Stanford Hypnotic Susceptibility Scale^41^. During the hypnosis induction, which lasted about 20 min, participants were instructed to close their eyes, relax and breathe deeply. The experimenter confirmed that the participants were in trance via the first item of the Stanford Hypnotic Susceptibility Scale^41^. This item entails that the participants stretch out their right arm. Then, the arm gets heavy as if participants carry a heavy weight in their hand. When the hand moved downwards at least 15 cm, the item was scored as passed. Then, the experimenter suggested safety via the imagination of a safe place. She told participants that she takes them for a journey to a place where they feel completely safe. The feeling of safety was described as a warm feeling like being cuddled into a blanket. Figure 1 illustrates this suggestion with a participant imagining the blanket. The verbatim text and recording of the safety suggestion is available as supplementary online material in English and German.

After the suggestion of safety, the experimenter asked participants to open their eyes. Then, the participants played the two tasks including the delayed gratification task as described below. Between both tasks, the suggestion of safety was repeated to re-intensify the feeling. Each task lasted about 10 min. After completing the tasks, the participants were led out of the hypnotic state. In the end of the hypnosis condition, the experimenter asked the participants how strongly they experienced the feeling of safety. The scale ranged from 1 for “not at all” to 5 for “I felt very safe”. After answering this question, participants filled in the Inventory Scale of Hypnotic Depth (ISHD^42^). We obtained a Cronbach’s alpha of 0.86 in our sample, replicating findings of Riegel and colleagues^42^.

#### Delayed gratification task

**Figure 2:**
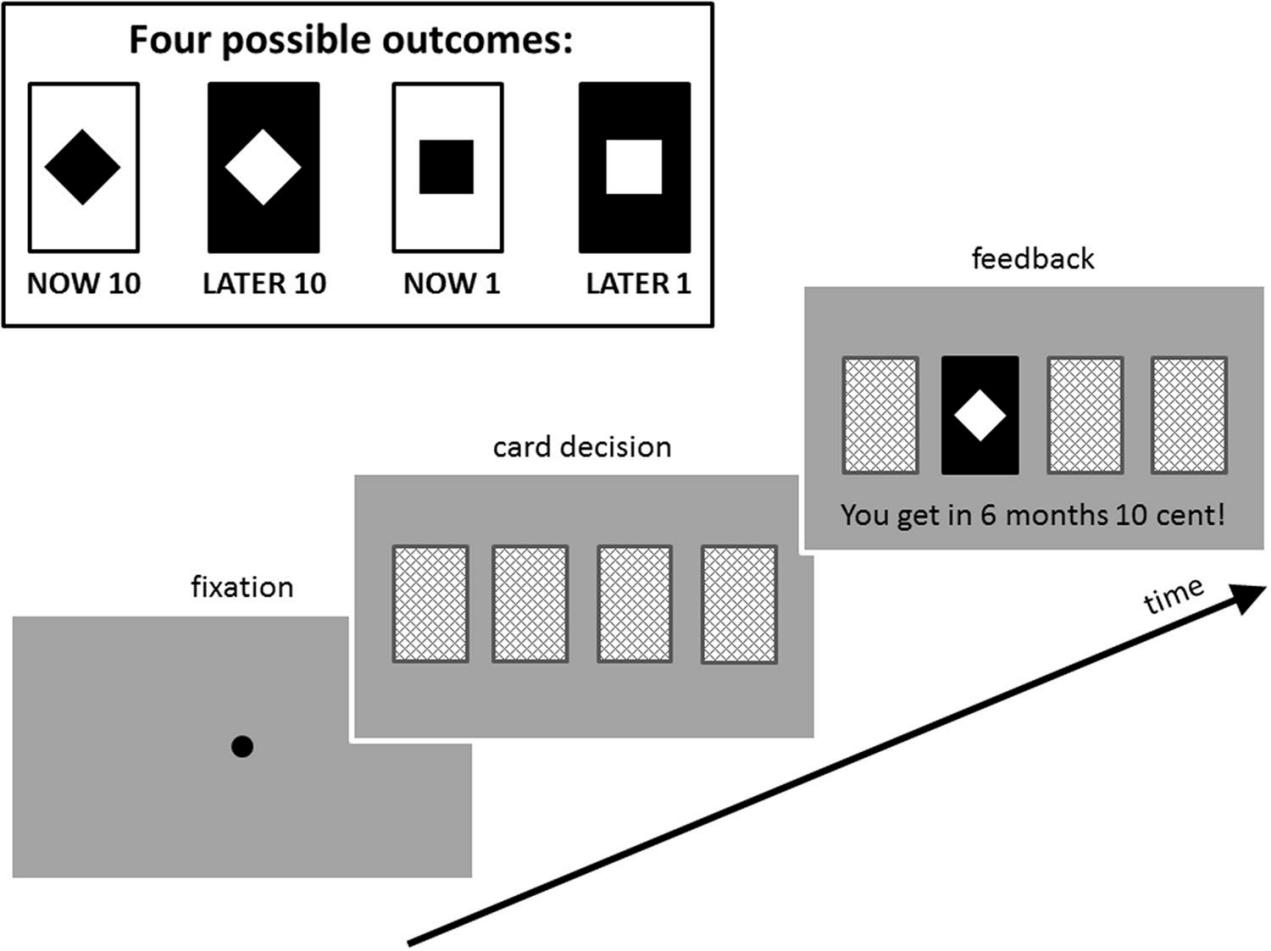
Task design. Four possible outcomes in the delayed gratification task (upper part) and a schematic illustrating the time-course of one trial of the task (lower part).

Participants engaged in the delayed gratification task described in Schmidt et al.^27^, which consisted of 125 experimental trials in each of the two conditions. At the beginning of each trial, a fixation dot was presented on a computer screen for a random interval between 300–700 ms (Fig. 2). Subsequently, an image representing the backs of four playing cards was presented. On each trial, participants chose one of the four card locations by pressing one of four corresponding buttons on their arm rest. They were instructed beforehand that each card choice would result in one of four possible outcomes, as indicated by one of two symbols presented on a contrasting background: 10 cents (diamond) or 1 cent (square) received either at the end of the experiment (white background) or after six months (black background). After a random interval between 300–700 ms following the response, the outcome of the selected card was shown together with the elaboration "You win now / in 6 months 10 / 1 cent!” (in German) as appropriate to the outcome. This feedback was displayed for 1500 ms. All stimuli in the delayed gratification task occupied about 6° of visual angle horizontally and 4° vertically. Please note that participants’ card choices did not affect the actual outcomes in this game, so the task does not measure delay discounting behavior. Instead, our delayed gratification task was optimized to obtain a stable number of trials for all outcomes to compute reliable ERP signals. Participants received each of the four outcomes 30 times in a pseudo-randomized order. Five additional trials were added to ensure that participants received different amounts of money across the two conditions. A feedback screen at the end of the task indicated how much money in total the participants would receive immediately and in six months.

#### Ratings and questionnaires

After completing the delayed gratification task, participants rated each outcome according to its valence and arousal on a 9-point rating scale with high values indicating positive valence and high arousal.

### EEG recording and ERP quantification

The EEG was recorded from 64 Ag/AgCl scalp electrodes, using two BrainAmp DC amplifiers (Brain Products GmbH, Gilching, Germany). Impedances were below 10 kΩ and electrode recordings were referenced to the electrode FCz online. The data were band-pass filtered during recording from 0.016 Hz to 250 Hz and sampled at 500 Hz. For offline data processing, EEGLAB^43^ running under the MATLAB environment (The MathWorks, Inc.) was used.

EEG artifacts were corrected using independent component analysis (ICA) as proposed by Debener et al.^44^. We removed eye-related artifact components by back-projection of all remaining components. The artifact-corrected data were then re-referenced to the mean of the voltages recorded at electrode locations TP9 and TP10. For ERP analysis, the data were low-pass filtered with 20 Hz (exact code in Matlab, using EEGLAB: EEG = pop_firws(EEG, 'fcutoff', 20, 'ftype', 'lowpass', 'wtype', 'blackman', 'forder', 1376, 'minphase', 0), segmented into epochs from − 200 ms to 800 ms around reward feedback onset, and baseline-corrected (− 200 ms to 0 ms). Epochs with residual artifacts were removed. Following the recommendation of Sambrook and Goslin^45^, who based a meta-analysis on published reward positivity waveforms, we evaluated the delay reward positivity during the period from 270 to 300 ms post-feedback (see darker grey area in Fig. 6). Reward positivity amplitude, averaged within this time window, was assessed at electrode FCz, where it reached maximum amplitude in agreement with the literature^20,22,23^. Following common practice, reward positivity amplitude was evaluated with a difference wave approach, which isolates variance in the ERP associated with reward trials while attenuating confounding ERP components such as the P300^45,46^. The computation of difference waves is generally recommended by Luck^47^, and is said to be especially appropriate as concerns the reward positivity^22,23^. In our previous study, we averaged across reward magnitude and computed a difference wave between the ERPs to later and immediate rewards, which we called the *delay reward positivity*^27^. We found that the delay reward positivity was significantly associated with impulsivity and self-control^27^. Note that larger reward positivity’s are indicated by more negative amplitudes, following the convention.

To test if the delay reward positivity is specifically affected by our experimental manipulation, we also quantified other components that play a role in reward processing. We quantified P2 amplitudes between 188 and 244 ms at electrode FCz^48^ (see lighter grey areas in Fig. 6). Also, we quantified P3 amplitudes between 328 and 380 ms at electrode Pz^49^.

## Results

### Subjective ratings of safety and hypnotic depth

After the induction of the hypnotic state in the hypnosis condition, participants were instructed to stretch out their right hand. Then, the experimenter suggested that this hand is getting very heavy as if they carried a heavy weight in it. The right hand of all participants moved downwards more than 15 cm after this suggestion, so all participants passed the hypnosis test item. The subjective ratings of participants indicated that they felt significantly safer in the hypnosis condition, *t*(23) = 25.0, *p* < 0.001, *d* = 5.1. Their mean safety rating was 4.1 (*SD* = 0.6) on a scale ranging from 1 for “not at all” to 5 for “I felt very safe”. Concerning hypnotic depth, which we measured via the ISHD with scores ranging from 36–144, participants had an average score of 99.3 (*SD* = 12.4), indicating a deep trance state according to Riegel et al.^42^. Suggestibility of participants, measured via the HGSHS score, was significantly associated with hypnotic depth, measured via the ISHD score, *r* = 0.42, *p* = 0.04. The more suggestible the participants were, the deeper was their hypnotic trance state.

### Delayed gratification task: behavior

**Figure 3:**
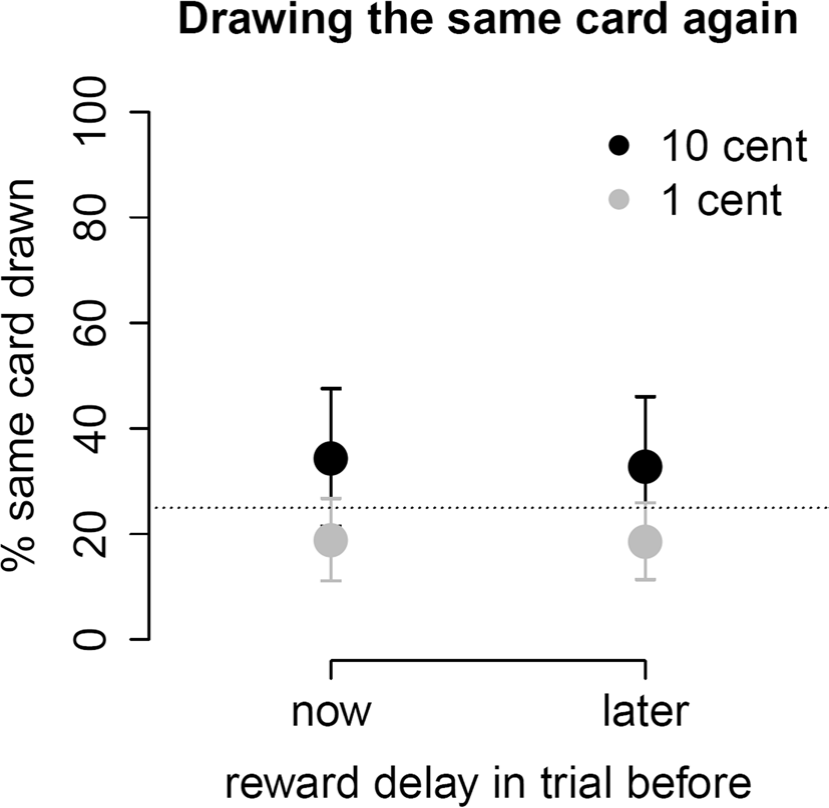
Participants more likely drew the same card again following large rewards, irrespective of the reward delay on the previous trial. The dotted line indicates chance probability (25%) for drawing the same card again. Error bars are 95% confidence intervals.

Response times for the card choices did not differ between conditions, *p* = 1. The mean response time to select a card was 770 ms (SD = 410 ms). Because the trial outcomes were delivered to the participants pseudo-randomly (unbeknownst to them), their card choices did not affect the outcomes. But we assumed that if participants drew the same card again more often after a good outcome than after a bad one, then that could be taken as evidence of adaptive behavior in response to the obtained outcomes. Participants could either draw the same card again or switch to another of the four cards, so the probability of drawing the same card again was 25%. An analysis of variance (ANOVA) on participants’ card choice behavior (same vs. different) with condition (hypnosis, control), reward magnitude on the previous trial (10 cents, 1 cent) and reward delay on the previous trial (now, later) as within-subject factors revealed a significant main effect of reward magnitude on participants’ card choice, *F*(1,23) = 7.5, *p* = 0.01, *η*_*G*_^2^ = 0.07. Following 10 cent rewards, participants drew the same card again on 34% of trials whereas following 1 cent rewards, they drew the same card again on only 19% of trials (Fig. 3). There were no significant main effects of condition or delay and no significant interaction effects.

### Delayed gratification task: ratings

**Figure 4:**
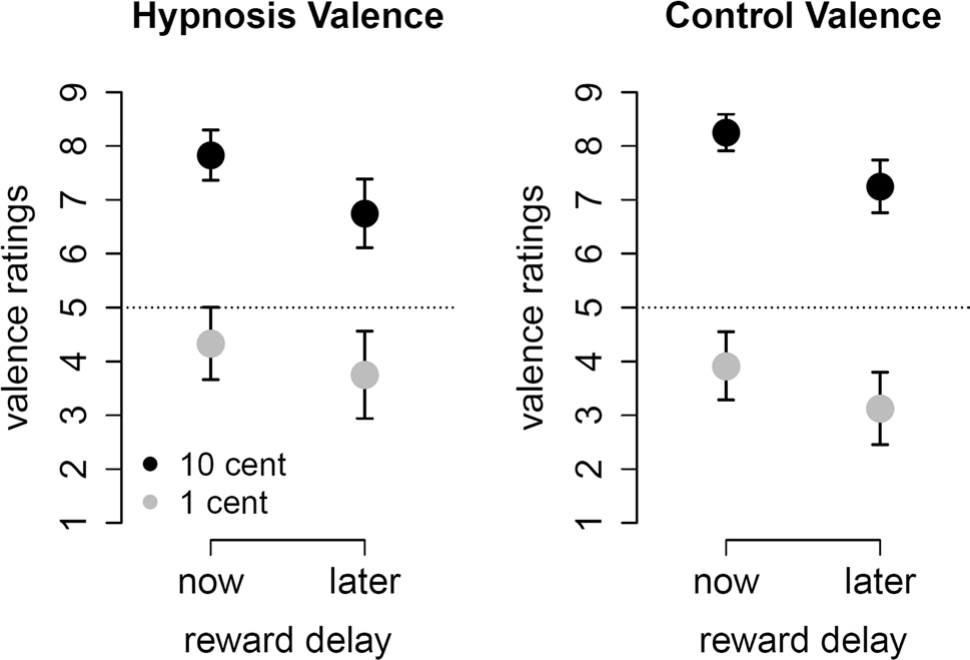
Participants rated higher and immediate rewards as more positive than lower and delayed rewards. In the hypnosis condition, participants rated higher rewards as less positive and lower rewards as less negative compared to the control condition. The dotted line indicates a neutral evaluation. Error bars are 95% confidence intervals.

After playing the delayed gratification task, participants rated the four outcomes concerning valence and arousal. We conducted an ANOVA with the within-subject factors condition (hypnosis, control), reward magnitude (10 cents, 1 cent) and reward delay (now, later) on the valence ratings of the previous trial and found a significant main effect of reward magnitude, *F*(1,23) = 133.2, *p* < 0.001, *η*_*G*_^2^ = 0.61 and a significant main effect of reward delay, *F*(1,23) = 14.5, *p* < 0.001, *η*_*G*_^2^ = 0.08. Figure 4 shows that participants rated higher magnitudes and immediate rewards as more positive than lower magnitudes and delayed rewards. Most importantly, we found a significant interaction of condition and reward magnitude, *F*(1,23) = 7.0, *p* = 0.01, *η*_*G*_^2^ = 0.03. Participants rated higher rewards as less positive and lower rewards as less negative in the hypnosis condition compared to the control condition, which is visible as a trend towards the neutral dotted line in Fig. 4.

**Figure 5:**
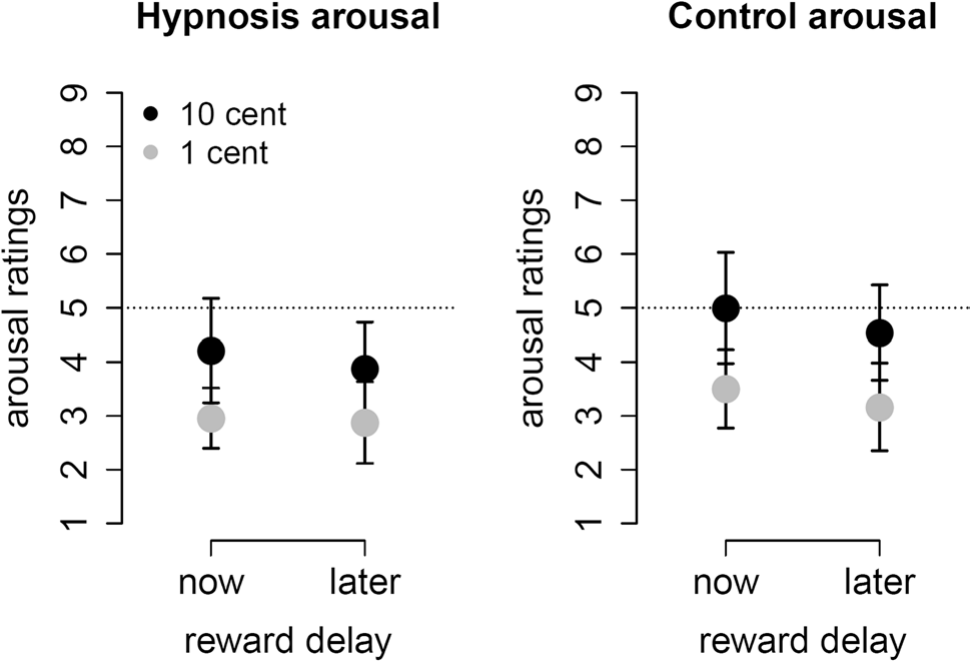
Participants rated higher rewards as more arousing than lower rewards. In the hypnosis condition, participants rated all rewards as less arousing than in the control condition. The dotted line indicates a neutral evaluation. Error bars are 95% confidence intervals.

A similar ANOVA on arousal ratings revealed a significant main effect of reward magnitude, *F*(1,23) = 8.7, *p* = 0.007, *η*_*G*_^2^ = 0.09 as well as a significant main effect of condition, *F*(1,23) = 4.5, *p* = 0.04, *η*_*G*_^2^ = 0.02. Figure 5 shows that participants rated higher rewards as more arousing. In the hypnosis condition, participants generally rated rewards as less arousing compared to the control condition.

### Delay reward positivity

The remainder of our analysis focuses on the delay reward positivity, which is determined by the difference in ERPs to feedback indicating immediate and delayed rewards averaged across reward magnitude^27^. This follows directly from the standard RewP approach in which confounding ERP components are removed by taking the difference between the raw ERPs to positive and negative outcomes^20,45,46^.

**Figure 6:**
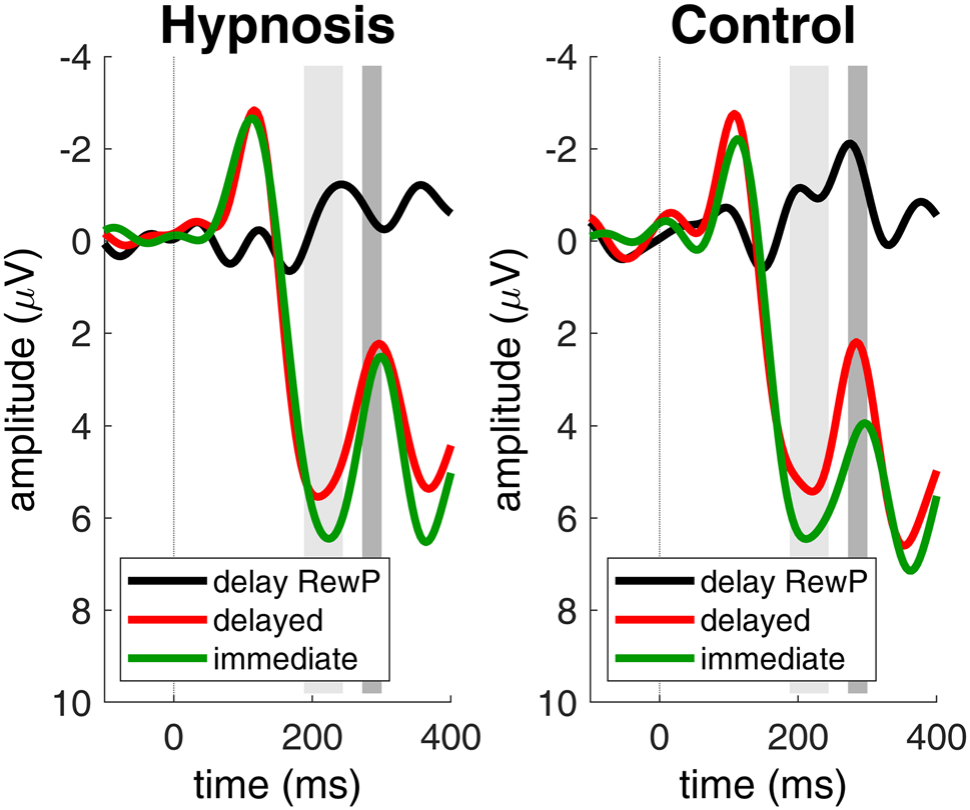
Delay reward positivity with associated ERPs averaged over reward magnitude. Participants showed significantly smaller reward positivity amplitudes in the hypnosis condition compared to the control condition (black lines). The lighter grey area indicates the P2 time window, the darker grey area indicates the delay reward positivity time window. Data were recorded at channel FCz. Negative is plotted up by convention.

**Figure 7:**
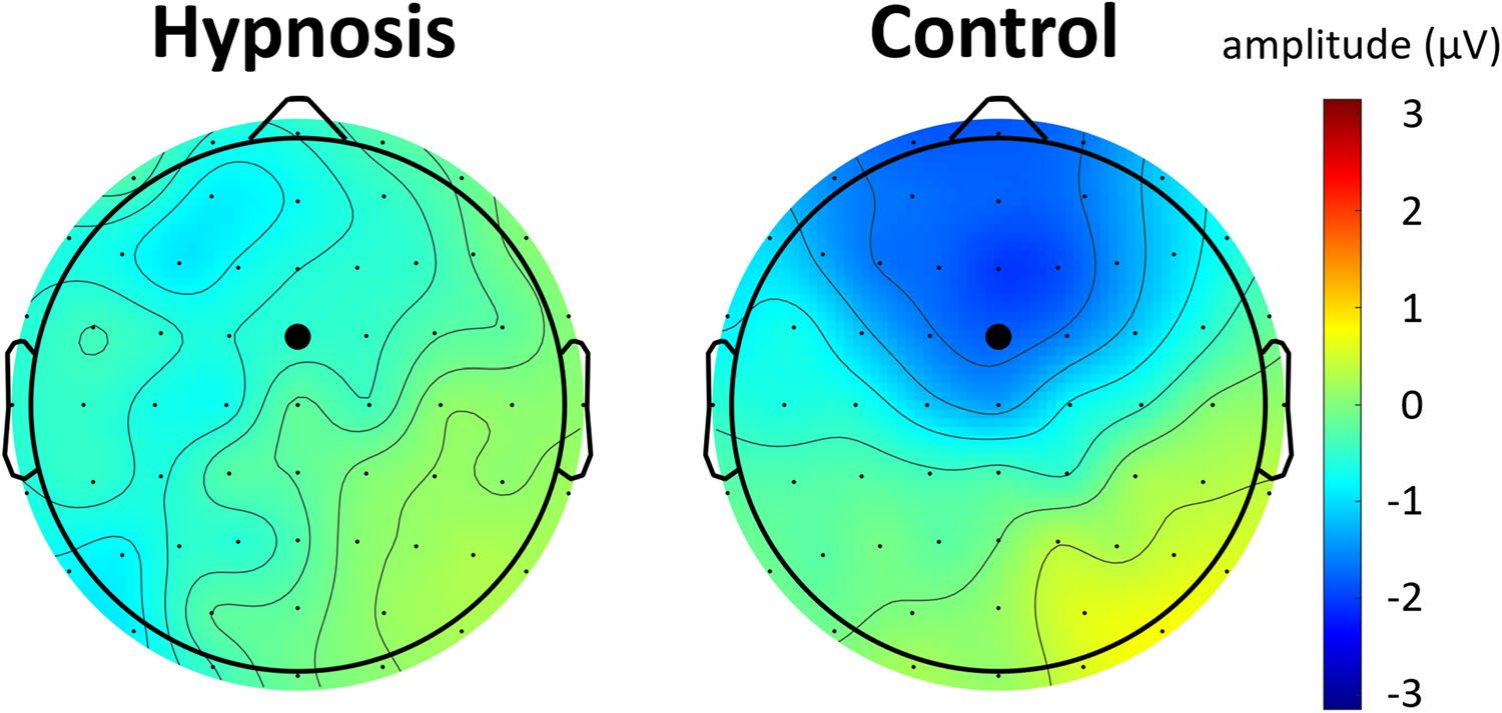
Topographical plot of the delay reward positivity averaged over reward magnitude during the delay reward positivity time window. Participants showed significantly smaller reward positivity amplitudes in the hypnosis condition compared to the control condition. Amplitudes were maximal over frontocentral electrode sites. The channel FCz is marked by a bigger black dot.

**Figure 8:**
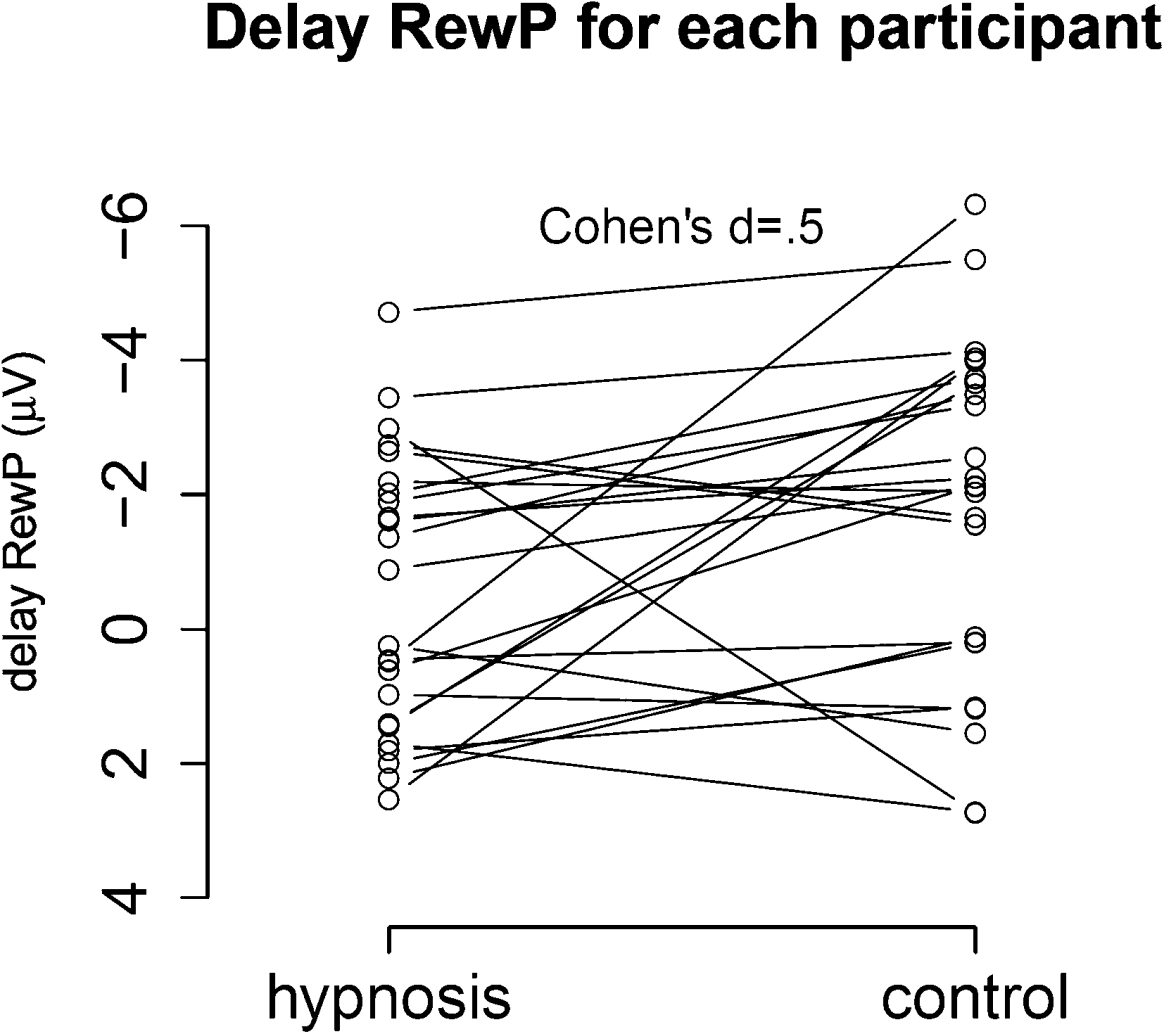
Delay reward positivity for each participant in both the hypnosis and control condition, illustrating the significant condition effect with Cohen’s d effect size.

**Figure 9:**
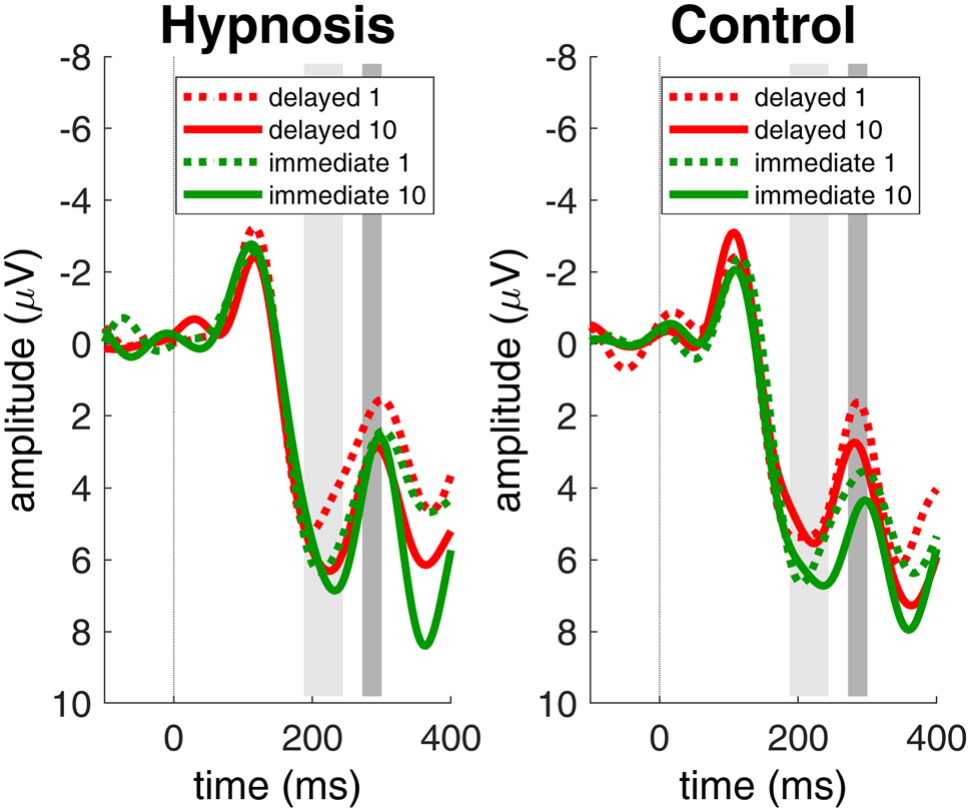
ERP responses to all four possible outcomes at FCz. For the delay reward positivity, the delayed outcomes (solid red and dotted red lines) and the immediate outcomes (solid green and dotted green lines) were aggregated over reward magnitude (1 or 10 cents). The lighter grey area indicates the P2 time window, the darker grey area indicates the delay reward positivity time window. Negative is plotted up by convention.

Figure 6 shows the delay reward positivity (black), and the associated raw ERPs to immediate (green) and delayed (red) rewards for the hypnosis condition and the control condition. Participants showed smaller delay reward positivity’s in the hypnosis condition compared to the control condition, *t*(23) = 2.3, *p* = 0.03, *d* = 0.5. Figure 7 shows the topographical maps of the reward positivity in both conditions with maximal reward positivity amplitudes over frontocentral electrodes, especially visible in the control condition. Figure 8 shows the reward positivity effect for every participant with consistently smaller reward positivity amplitudes in the hypnosis condition compared to the control condition. As a check, we also performed a 3-way ANOVA on raw ERP amplitudes that are shown in Fig. 9 with the within-factors condition (hypnosis, control), reward delay (immediate, delayed) and reward magnitude (1 cent, 10 cents). This ANOVA revealed a significant main effect of reward delay, *F*(1,23) = 9.2, *p* = 0.006, a significant main effect of reward magnitude, *F*(1,23) = 5.2, *p* = 0.03 and a significant interaction effect of condition and reward delay, *F*(1,23) = 5.1, *p* = 0.03. All other effects did not reach significance (*p* > 0.4). When added as a fourth factor in the ANOVA, the order of conditions did not affect the results significantly.

To test if the hypnosis and control conditions selectively affected the delay reward positivity, we also analyzed P2 and P3 amplitudes, which respectively occurred before and after the delay reward positivity. An ANOVA with the factors condition (hypnosis, control), reward delay (immediate, delayed) and reward magnitude (1 cent, 10 cents) on P2 amplitudes revealed a significant main effect of reward delay, *F*(1,23) = 11.7, *p* = 0.002. Figure 6 shows that the P2 amplitudes in the lighter grey area are more positive for immediate rewards than for delayed rewards. All other effects did not reach significance. A similar analysis of variance on P3 amplitudes revealed a significant main effect of reward magnitude, *F*(1,23) = 29.2, *p* < 0.001, revealing that higher rewards elicited higher P3 amplitudes than smaller rewards (Supplementary Figure). All other effects did not reach significance.

## Discussion

In this study, we show that participants who felt safer after a hypnotic suggestion showed reduced neuronal signals of delay discounting. After participants were successfully hypnotized and received a suggestion to feel safe, their delay reward positivity – the difference between the brain responses to immediate and delayed rewards – was significantly smaller than in the control condition. That observation indicates that the natural tendency to devalue future rewards was reduced. As this tendency is more pronounced in highly impulsive and less self-controlled participants^27^, we conclude that a state-induced feeling of safety made participants less impulsive and more self-controlled. We suggest that the induced feeling of safety was associated with an increased perception of reward certainty, which in turn reduced delay discounting.

The reduced delay reward positivity amplitude after the induction of safety parallels our previous finding of smaller delay reward positivity amplitudes in individuals with low impulsivity and high self-control as compared to individuals with high-impulsivity and low-self-control^27^. In that study, it is possible that the participants in the low impulsive and high self-control group just happened to feel relatively safe on the day of testing, as compared to participants in the high impulsive and low self-control group. As we did not collect data on participants’ feeling of safety in our previous study, we cannot test this. That said, our results indicate that the current emotional state participants are in when they are confronted with a delay discounting task has a great impact on how they respond to it.

It is important to differentiate between two possible interpretations of the data. The first interpretation is that participants who felt safe in the hypnosis condition differentiated less between immediate and delayed rewards. The second interpretation is that participants who felt safe in the hypnosis condition were just very relaxed and therefore did not care about any rewards. To address this, we first evaluate the neuronal and behavioral responses that could indicate a general loss of involvement in the hypnosis condition. In participants’ brain responses, we see that the delay reward positivity was reduced in the hypnosis condition, while the amplitudes of other prominent ERP components – namely the P2 and P3 – were similar in both conditions. This can be interpreted as evidence against the second interpretation, as ERP amplitudes were not generally reduced in the hypnosis condition. Participants’ card choices show that they chose the same card more often after receiving a higher reward in both conditions, indicating that they adapted their behavior according to the obtained outcome in the trial before in both conditions. Also, response times were similar in both conditions, showing that participants in the hypnosis condition responded as fast as in the control condition. This shows a certain involvement in the delay discounting game, contradicting the second interpretation. On the other hand, the valence ratings of participants show a clear tendency towards the neutrality line in Fig. 4, which is an argument for the second interpretation. Although we cannot rule out the second interpretation, most of the evidence favors the first interpretation, namely that participants who felt safe in the hypnosis condition differentiated less between immediate and delayed rewards.

The results are in line with previous results showing that children are more likely to wait for a delayed reward when the experimenter is reliable^1,11^. The perception that the person delivering the delayed reward is reliable is associated with increased certainty that the reward will be delivered, which in turn is associated with a feeling of safety. Instead of manipulating the reliability of the reward deliverer, we developed an intervention to increase this feeling of safety directly.

We conclude that the suggestion of safety can serve as a potential intervention in disorders that are associated with impulsivity and a lack of self-control, such as in substance use disorder. As we tested highly suggestible participants in our study, it is important to generalize the results also to lower suggestible participants. Suggestibility is a stable trait with a 25-year retest reliability of *r* = 0.7^50^. It has been shown that certain substances increase suggestibility such as alcohol^51^, cannabis^52^ and LSD^53^. If individuals with substance abuse show higher suggestibility because of the substance they are consuming or as a pre-existing stable personality trait has not been investigated yet. The fact that individuals with smartphone addiction show higher suggestibility scores^54^ might be an indicator of higher trait suggestibility in addiction-prone individuals without consuming a substance that increases suggestibility. As noted above, people with disorders that go along with low levels of self-control and high levels of impulsivity like drug addiction and gambling show increased delay discounting^6–8^. Reducing delay discounting indicated by lower delay reward positivity amplitudes is associated with positive outcomes like lower impulsivity and more self-control^27^. Therefore, it may be possible to hypnotize impulsive and low self-controlled people with the suggestion to feel safe, thereby reducing their tendency to devalue future rewards. Promising examples from smoking cessation studies show that hypnotic interventions lead to high rates of long-term abstinence in substance abuse^55,56^. To establish long-term effects, it is possible to repeat this procedure several times or to use post-hypnotic suggestions where the feeling of safety is associated with a trigger that elicits the feeling of safety after the hypnotic state is over.

## Supplementary Information

Supplementary Information 1.

Supplementary Audio 1.

Supplementary Information 2.

Supplementary Audio 2.

Supplementary Information 3.
