# Supplementary material for: Hypnotic suggestions of safety reduce neuronal signals of delay discounting
Source: Sci Rep. 2021 Feb 1;11:2706. doi: 10.1038/s41598-021-81572-2 (PMC7851403; doi:10.1038/s41598-021-81572-2)
Supplement: Supplementary file 5 — Supplementary Information 3. [file 41598_2021_81572_MOESM5_ESM.docx]

**Supplementary Material**

Hypnotic suggestions of safety reduce neuronal signals of delay discounting

Authors:

Barbara Schmidt^1^

Clay B. Holroyd^2^

Affiliation:

^1^Institute of Psychology, University of Jena, Jena, Germany

^2^Department of Experimental Psychology, Ghent University, Ghent, Belgium

Full contact information of the corresponding author:

Dr. Barbara Schmidt

Am Steiger 3, Haus 1

07743 Jena, Germany

e-Mail: schmidt.barbara@uni-jena.de

phone: +49 6341 9 45149

fax: +49 6341 9 45142


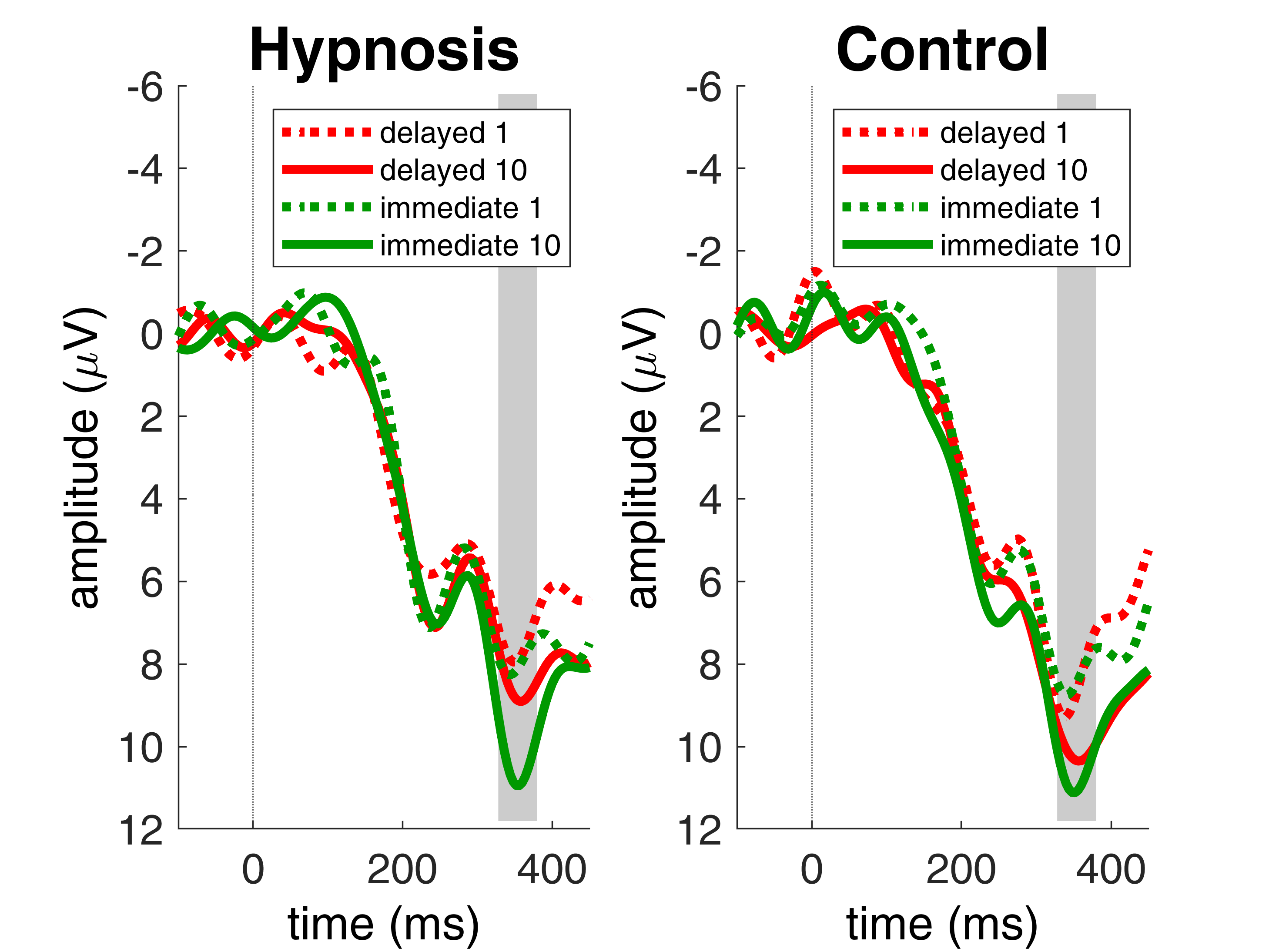


*Supplementary Figure:* ERP responses to all four possible outcomes (delayed 1 cent; delayed 10 cents; immediate 1 cent; immediate 10 cents) at Pz. The grey area indicates the P3 analysis time window. Higher reward magnitudes (solid lines) elicited significantly higher P3 amplitudes than smaller reward magnitudes (dotted lines). Negative is plotted up by convention.
